# Displacement-Based Back-Analysis of the Model Parameters of the Nuozhadu High Earth-Rockfill Dam

**DOI:** 10.1155/2014/292450

**Published:** 2014-07-08

**Authors:** Yongkang Wu, Huina Yuan, Bingyin Zhang, Zongliang Zhang, Yuzhen Yu

**Affiliations:** ^1^State Key Laboratory of Hydroscience and Engineering, Department of Hydraulic Engineering, Tsinghua University, Beijing 100084, China; ^2^HydroChina Kunming Engineering Corporation, Kunming 650051, China

## Abstract

The parameters of the constitutive model, the creep model, and the wetting model of materials of the Nuozhadu high earth-rockfill dam were back-analyzed together based on field monitoring displacement data by employing an intelligent back-analysis method. In this method, an artificial neural network is used as a substitute for time-consuming finite element analysis, and an evolutionary algorithm is applied for both network training and parameter optimization. To avoid simultaneous back-analysis of many parameters, the model parameters of the three main dam materials are decoupled and back-analyzed separately in a particular order. Displacement back-analyses were performed at different stages of the construction period, with and without considering the creep and wetting deformations. Good agreement between the numerical results and the monitoring data was obtained for most observation points, which implies that the back-analysis method and decoupling method are effective for solving complex problems with multiple models and parameters. The comparison of calculation results based on different sets of back-analyzed model parameters indicates the necessity of taking the effects of creep and wetting into consideration in the numerical analyses of high earth-rockfill dams. With the resulting model parameters, the stress and deformation distributions at completion are predicted and analyzed.

## 1. Introduction

A large number of high earth-rockfill dams located in western China with heights of 250 m to 300 m are currently under construction or being planned. Among these dams, the Nuozhadu earth-rockfill dam, with a height of 261.5 m, is the highest earth-core rockfill dam under construction in China. To ensure safety, a large number of observation instruments have been installed at different elevations and different zones in the dam during the construction period. To date, field observation data have been collected to analyze the characteristics of dam materials and the stress-deformation distribution in the dam and to facilitate the prediction of future deformation.

The calculation of earth-rockfill dam deformation is affected by many factors, such as the representativeness of soil samples, the size effect of laboratory tests, the differences of sample preparation and loading conditions from the real construction conditions, and the imperfection of the constitutive model and the numerical method. Moreover, along with the construction process, the model parameters of dam materials will change with time due to the breakage and wetting of rockfill particles. Therefore, it is of great importance to dynamically back-analyze the model parameters of dam materials based on field observation data to improve the accuracy of deformation prediction.

Displacement back-analysis is an effective method to identify the model parameters of soils and rocks. In conventional back-analysis methods, the optimal values of parameters are usually progressively approximated by minimizing the error function through iterations. In general, the range and initial values of the parameters should be given before the analysis, the time-consuming finite element method (FEM) calculation is performed frequently, the rate of convergence is slow, and sometimes the back-analysis fails for large-scale nonlinear problems. Furthermore, the result is often affected by the initial values and a local minimum or premature convergence is likely to be obtained. Therefore, for large-scale multiparameter nonlinear problems, the solution is sometimes unstable. In recent years, the artificial intelligence back-analysis method was introduced to geotechnical engineering. With the development of intelligent optimization algorithms, the artificial intelligence back-analysis method is continuously further improved. Extensive studies have been conducted to develop different displacement-based back-analysis methods [1–8]. Among them, intelligent back-analysis methods based on artificial neural networks and generic algorithms have been utilized and shown great potential in geotechnical engineering. In these methods, the strong nonlinear relationship between the physical quantities (e.g., displacement, seepage, and water pressure) and the unknown parameters can be mapped well with artificial neural networks. The drawback of frequently calling the time-consuming finite element (FE) analysis in the process of optimization can be overcome by replacing FEM calculation with trained neural networks. Premature convergence can be avoided and a global optimal solution can be acquired by employing evolutionary algorithm instead of conventional optimization methods.

To date, studies of displacement-based back-analysis methods have mainly focused on underground engineering and rock mass, whereas studies on dam projects are relatively scarce. In addition, there have been few studies on the back-analyses of dams in the process of construction, which are usually performed after the construction is completed. In addition, the displacement-based back-analyses, usually focused on the constitutive model parameters, pay little attention to the parameters of wetting and creep models. In particular, currently, there are no research results concerning the back-analysis of wetting deformation in geotechnical engineering.

Wetting deformation and creep deformation, for which many numerical calculation models and methods have been built, have great significance in the stress redistribution and stability of earth-rockfill dams. The mechanism of wetting deformation is generally investigated using laboratory tests [9–12], whereas the mechanism of creep deformation is difficult to study in a laboratory [13–15]. The reason is that a long loading time is needed in creep tests, which is almost impossible for rockfill materials in large-scale triaxial testing facilities. Therefore, back-analysis based on deformation observation data appears to be the only feasible study method. In high earth dams, the internal stress is high. In addition, the particles of materials experience obvious breakage, and large shear deformation exists, which results in the stress and deformation behaviors of high earth dams being significantly different from those of low dams. The loading deformation, creep deformation, and wetting deformation are simultaneous in the process of construction and impounding. Thus, they should all be considered in the numerical analysis when accurately simulating characteristics. The back-analysis of model parameters is important for studying the behaviors of high earth-rockfill dams and further verifying the effectiveness of the models.

Nuozhadu dam is the first high earth-rockfill dam with comprehensive monitoring. The collected observation data of Nuozhadu dam fully reflect the state of the dam and are of great significance for the study of the stress and deformation characteristics in high earth-rockfill dams. In this study, an intelligent back-analysis method based on artificial neural networks and evolutionary algorithm [16, 17] is employed to back-analyze the model parameters of the dam using selected field monitoring displacement data. The parameters of the constitutive model, as well as the wetting and creep models, are back-analyzed all together. In this method, time-consuming finite element analysis is replaced by an artificial neural network with optimal structure trained by an evolutionary algorithm, and the model parameters are also optimized using an evolutionary algorithm. To avoid the construction of a large number of training samples associated with simultaneous back-analysis of the three main dam materials, the back-analyses of different dam materials are conducted separately and sequentially according to material zoning, construction process, and observation point locations. Along with the construction process, displacement back-analyses were performed at different construction stages to reveal the evolution of dam material properties. The numerical results calculated using back-analyzed model parameters are in good agreement with the monitoring data. The revealed evolution of dam material properties has been partially verified by field examination results. The calculation results based on different sets of back-analyzed parameters were compared to investigate the influence of creep and wetting deformations. With the newly obtained parameters, the stress and deformation distributions at completion were predicted and analyzed.

## 2. Project Description

The Nuozhadu hydropower station is located on the main stream of the lower Lancang River, near Pu'er City of Yunnan Province. The total installed capacity is 5,850 MW, and the designed annual average power output is 239 × 10^8^ kW·h. This project is composed of the earth-core rockfill dam, an open spillway on the left bank, flood discharge tunnels, and underground water diversion structures. The earth-core rockfill dam has a maximum height of 261.5 m, and the total storage capacity of the reservoir is 237 × 10^8^ m^3^. The dam is now close to its completion.

**Figure 1:**
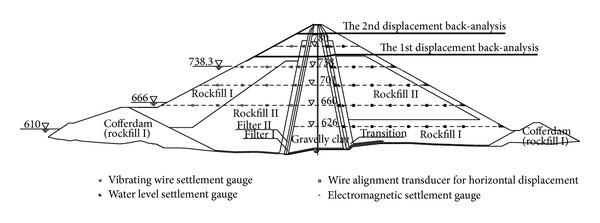
The maximum cross-section of Nuozhadu earth-core rockfill dam.

The maximum cross-section of the dam, with material zoning, is shown in Figure 1. There are six types of dam materials in total, among which rockfill I, rockfill II, and gravelly clay (the core material) are the three main dam materials. To control the deformation of the dam, rockfill materials with high deformation moduli are used in the main rockfill zones, and to reduce the differential deformation between the core wall and the rockfill, a certain amount of gravel is mixed into clay for use as an impervious core material.

**Figure 2:**
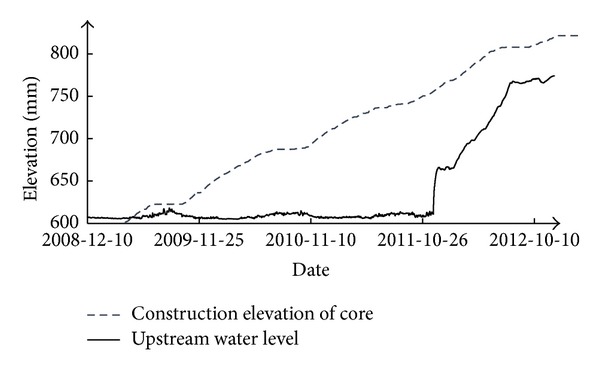
Construction process of the dam and water level of the reservoir.

The construction process of the core and the impounding process of the reservoir are shown in Figure 2. Dam construction was started in January 2008 and completed at the end of December 2012. During the construction period, the filling of the dam was shut down in the flood season every year. The upstream water level was stabilized at approximately 605 m before December 2011. Then, the water level rose with the impounding of the reservoir. At the end of December 2012, the upstream water level reached 774 m.

Observation instruments were installed on several cross-sections of the dam. The layout of the observation instruments on the maximum cross-section is shown in Figure 1. Vibrating wire settlement gauges were installed in the upstream rockfill zones at 4 elevations. Water level settlement gauges and wire alignment transducers for horizontal displacement were installed in the downstream rockfill zones at 5 elevations. In the core wall of gravelly clay, there were electromagnetic settlement gauges every 3 m of height.

## 3. Material Models and Displacement Back-Analysis Method 

### 3.1. Material Models

The stress-strain relationship, creep behavior, and wetting deformation of the dam materials are needed to simulate the behavior of the dam during the construction and impounding process. Duncan and Chang's E-B model [18] was used to describe the stress-strain relationship. It is a nonlinear elastic model and has been widely used in geotechnical engineering, especially in the numerical analyses of earth dams. A seven-parameter creep model [19] and a modified Shen's three-parameter wetting model [20] were used to describe the creep behavior and wetting deformation of the dam materials, respectively. A brief introduction of the Duncan and Chang's E-B model, the creep model, and the wetting model is provided below.

#### 3.1.1. Duncan and Chang's E-B Model

This model was developed to provide a simple framework encompassing the most important characteristics of soil stress-strain behavior. The nonlinear stress-strain curves are represented by hyperbolae, whose instantaneous slope is the tangent modulus, *E*_*t*_. And the tangent modulus *E*_*t*_ can be expressed as follows:

(1) $$\begin{array}{c} E_{t}={\left[1-\frac{R_{f}\left(1-\sin\phi \right)}{2c\cdot \cos\phi +2\sigma_{3}\cdot \sin\phi }\left(\sigma_{1}-\sigma_{3}\right)\right]}^{2}K\cdot P_{\text{a}}{\left(\frac{\sigma_{3}}{P_{\text{a}}}\right)}^{n}, \end{array}$$

where *K*, *n*, *c*, *ϕ*, *R*_*f*_, *σ*_3_, and *P*_a_ are modulus number, modulus exponent, cohesion intercept, friction angle, failure ratio, minor principal stress, and atmospheric pressure, respectively.

The bulk modulus can be expressed as

(2) $$\begin{array}{c} B=K_{b}\cdot P_{\text{a}}{\left(\frac{\sigma_{3}}{P_{\text{a}}}\right)}^{m}, \end{array}$$

where *K*_*b*_ is bulk modulus number and *m* is bulk modulus exponent.

The Mohr-Coulomb envelopes for almost all soils are curved to some extent, and the wider the range of pressure involved the greater the curvature, especially for cohesionless soils such as sand, gravel, and rockfill. For example, in the bottom near the center of a large dam, rockfill may be confined under such a large pressure that the friction angle may be several degrees smaller than that near the surface of the slopes. This variation in property may be represented by an equation of the form

(3) $$\begin{array}{c} \phi =\phi_{0}-\Delta \phi \log\left(\frac{\sigma_{3}}{P_{\text{a}}}\right), \end{array}$$

where *ϕ*_0_ is the value of *ϕ* for *σ*_3_ = *P*_a_ and Δ*ϕ* is the reduction in *ϕ* for a 10-fold increase in *σ*_3_.

It can be seen that there are seven parameters in Duncan and Chang's E-B model, that is, *c*, *ϕ* (or *ϕ*_0_, Δ*ϕ*), *R*_*f*_, *K*, *n*, *K*_*b*_, and *m*, which can be evaluated using a group of conventional triaxial tests.

#### 3.1.2. Seven-Parameter Creep Model

The seven-parameter creep model is commonly used in the numerical analyses of earth dams. Merchant's equation is used to describe the rheological deformation curve in the creep model:

(4) $$\begin{array}{c} \varepsilon \left(t\right)=\varepsilon_{i}+\varepsilon_{f}\left(1-e^{-\alpha t}\right), \end{array}$$

where *ε*_*i*_ is the instantaneous deformation, *ε*_*f*_ is the final creep deformation, and *α* is the index of rheological decay over time. When differentiating (4) with respect to time, the strain rate is obtained as

(5) $$\begin{array}{c} {\dot{\varepsilon }}_{t}=\alpha \varepsilon_{f}e^{-\alpha t}=\alpha \left(\varepsilon_{f}-\varepsilon_{t}\right), \end{array}$$

where *αε*_*f*_ is the initial strain rate. The strain rate ${\dot{\varepsilon }}_{t}$ can be divided into two parts, the volumetric strain rate ${\dot{\varepsilon }}_{vt}$ and the shear strain rate ${\dot{\gamma }}_{t}$, which are expressed as follows:

(6) $$\begin{array}{c} {\dot{\varepsilon }}_{vt}=\alpha \left(\varepsilon_{vf}-\varepsilon_{vt}\right), \end{array}$$

(7) $$\begin{array}{c} {\dot{\gamma }}_{t}=\alpha \left(\gamma_{f}-\gamma_{t}\right), \end{array}$$

where *ε*_*vf*_ is the final volumetric deformation and *γ*_*f*_ is the final shear deformation. Some studies have demonstrated that *ε*_*vf*_ is related to the confining pressure *σ*_3_ and the generalized shear stress *σ*_*s*_ whereas *λ*_*f*_ is related to the stress level *S*_*l*_ with a nonlinear relationship. Here, they are assumed to be

(8) $$\begin{array}{c} \varepsilon_{vf}=b{\left(\frac{\sigma_{3}}{Pa}\right)}^{m_{c}}+\beta {\left(\frac{\sigma_{s}}{Pa}\right)}^{n_{c}}, \end{array}$$

(9) $$\begin{array}{c} \gamma_{f}=d{\left(\frac{S_{l}}{1-S_{l}}\right)}^{L_{c}}, \end{array}$$

where *Pa* is the atmospheric pressure. Using the Prandtl-Reuss flow rule, the strain rate tensor can be expressed as

(10) $$\begin{array}{c} \left\{\dot{\varepsilon }\right\}=\frac{1}{3}{\dot{\varepsilon }}_{vt}I+{\dot{\gamma }}_{t}\frac{\left\{s\right\}}{\sigma_{s}}, \end{array}$$

where {*s*} is the deviator stress tensor and *I* is second-order identity tensor.

Overall, there are seven parameters in the creep model [(8), (9), and (10)], that is, *α*, *b*, *d*, *m*_*c*_, *β*, *n*_*c*_, and *L*_*c*_.

#### 3.1.3. Modified Shen's Three-Parameter Wetting Model

A modified Shen's three-parameter wetting model was used to calculate the wetting deformation of the dam materials. The wetting deformation in the model consists of two components, the volumetric wetting deformation *ε*_*vs*_ and the shear wetting deformation *γ*_*s*_. They are related to the confining pressure *σ*_3_ and the stress level *S*_*l*_, respectively, as follows:

(11) $$\begin{array}{c} \varepsilon_{vs}=\frac{\sigma_{3}}{a_{w}+b_{w}\sigma_{3}}, \end{array}$$

(12) $$\begin{array}{c} \gamma_{s}=c_{w}\cdot \frac{S_{l}}{1-S_{l}}. \end{array}$$

Using the Prandtl-Reuss flow rule, the strain tensor can be expressed as follows:

(13) $$\begin{array}{c} \left\{\varepsilon \right\}=\frac{\varepsilon_{vs}}{3}I+\frac{\gamma_{s}}{\sigma_{s}}\left\{s\right\}. \end{array}$$

There are three parameters, that is, *a*_*w*_, *b*_*w*_, and *c*_*w*_, in the wetting model, which can be obtained by laboratory tests.

### 3.2. Displacement Back-Analysis Method Based on Artificial Neural Network and Evolutionary Algorithm

Because of the complexity of geotechnical engineering problems, conventional parameter back-analysis methods often require a large number of forward finite element analyses and thus a long computation time, and the result may be easily trapped in local minimum values. In the back-analysis method [17] applied here, an artificial neural network, with strong nonlinear mapping ability, is trained to simulate the relationship between model parameters and displacement response to reduce the time of forward analysis. And an evolutionary algorithm with global convergence is used to train the network and optimize the model parameters. Prior to the training of the artificial neural network, the total number of hidden nodes should be specified. The structure of the neural network (i.e., dividing the hidden nodes into different number of hidden layers) and the parameters for each node were initialized arbitrarily and optimized using the evolutionary algorithm.

**Figure 3:**
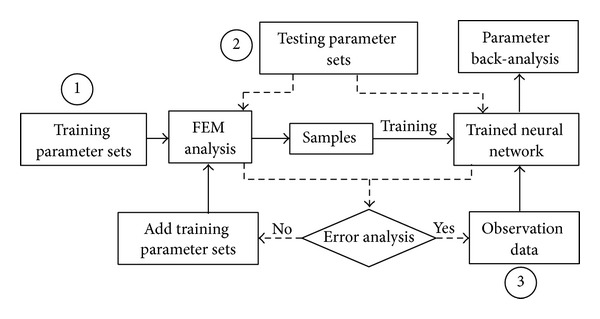
Displacement back-analysis method based on neural network and evolutionary algorithm.

Figure 3 shows the flowchart of the back-analysis method, including three main steps: (1) performing forward FEM analysis on the training parameter sets to generate samples and then using evolutionary algorithm and Vogl's algorithm to train and optimize the neural network; (2) constructing testing parameters sets randomly, and accessing the accuracy of the trained neural network for the testing parameters sets by comparing its results with that of the FEM analysis, and if the error criterion is not satisfied, adding several training parameter sets, and then going back to step (1) to optimize and train the neural network again until the error criterion is met; and (3) optimizing the soil parameters using evolutionary algorithm, observation data and the trained neural network.

The displacement back-analysis software EBA-EANN, developed based on this method, has been successfully applied to several earth-rockfill dams in China with good results [21].

## 4. Back-Analysis of Model Parameters

### 4.1. FEM Model

**Figure 4:**
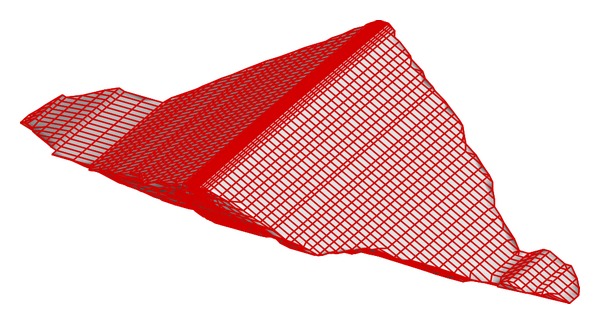
3D FEM mesh of Nuozhadu earth-core rockfill dam.

An FEM model was used to calculate the stress and deformation response of the dam and to generate the training samples for the neural network.  Figure 4 shows the 3D FEM mesh, which contains 20,663 elements. For the constructed part, the actual construction process was simulated, whereas for the remaining part, the designed construction process was simulated. The stress-strain relationship, creep behavior, and wetting deformation of the dam materials are described using Duncan and Chang's E-B model [18], the seven-parameter creep model [19], and a modified Shen's three-parameter wetting model [20], respectively. And the computational time using the FEM model to perform a static calculation for one of the training samples is about 20 minutes.

### 4.2. Stepwise Displacement Back-Analyses

**Figure 5:**
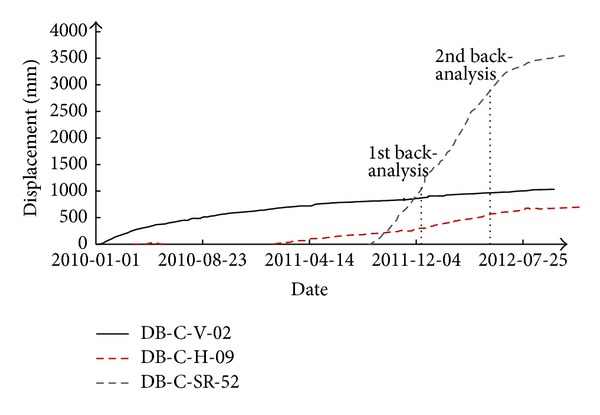
Measured displacements at typical observation points.

Displacement back-analyses were performed at two different construction stages (see Figure 1), and the details are listed in Table 1. For each back-analysis, displacement increases during observation periods with reasonable data were used, and only the model parameters of the three main dam materials were back-analyzed. The displacements at a few typical observation points inside the dam are plotted in Figure 5, where the displacements increase with the construction of the dam and display reasonable variations.

#### 4.2.1. The 1st Displacement Back-Analysis

With sensitivity analysis of the model parameters, only the four main E-B model parameters, that is, *K*, *n*, *K*_*b*_, and *m*, were back-analyzed during the first back-analysis, and the creep and wetting deformations were not considered. The parameters obtained by laboratory tests are listed in Table 2. For simultaneous back-analysis of the three main dam materials, the number of parameters is 3 × 4 = 12. The number of training samples is 3^12^≈ 5 × 10^5^ when taking 3 values for each parameter and constructing the samples by way of full factorial design. The computation cost would be too high.

To avoid this problem, the back-analyses of different dam materials were decoupled by considering material zoning, construction process, and observation point locations. First, most of the water level settlement and wire alignment transducer observation points at EL. 626 m are in the downstream rockfill I zone. As this region is at the bottom of the dam and was constructed at an earlier time, the displacement distribution in this region mainly depends on the model parameters of rockfill I, whereas other materials with given density act as loading on this region. Therefore, the model parameters of rockfill I could be back-analyzed separately from displacement measurements in the rockfill I zone at EL. 626 m. Then, with the obtained model parameters of rockfill I, the model parameters of rockfill II could be back-analyzed from displacement measurements at EL. 660 m and EL. 701 m, except for the measurements in the core wall. Finally, the model parameters of gravelly clay could be back-analyzed using the settlement measurements of electromagnetic gauges in the core wall. With this treatment, the number of samples was reduced to 3^4^ × 3 = 243, which is much lower than the simultaneous back-analysis number.

**Figure 6:**
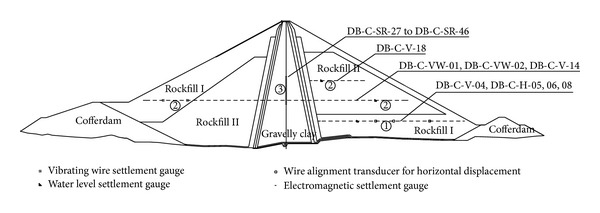
The measurement points used in the 1st back-analysis.

Reasonable observation data of selected measurement points were used as the targets of back-analysis. The measurement points were selected on the basis of previous numerical analyses, quality of observation data, and experiences of numerical calculation. The locations of the measurement points used in the 1st back-analysis are shown in Figure 6. First, the model parameters of rockfill I were back-calculated from the observation data of DB-C-H-05, DB-C-H-06, DB-C-H-08, and DB-C-V-04. At this time, the model parameters of rockfill II and gravelly clay took laboratory test values. Then, the model parameters of rockfill II were back-calculated from the observation data of DB-C-VW-01, DB-C-VW-02, DB-C-V-14, and DB-C-V-18. At last, the model parameters of gravelly clay were back-calculated from the observation data of DB-C-SR-27 to DB-C-SR-46.

**Figure 7:**
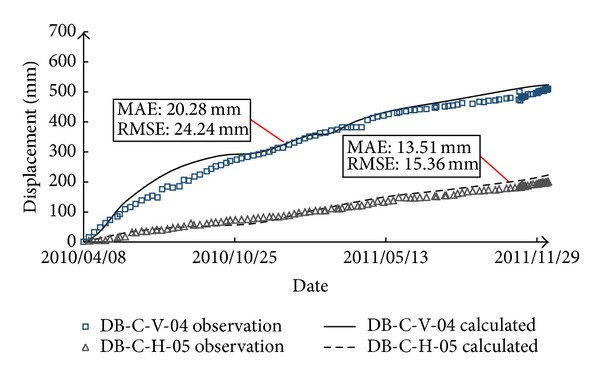
Comparison between calculated results (1st back-analysis parameters) and observation data.

The results of the 1st back-analysis are also listed in Table 2, from which it can be seen that the back-calculated model parameters of rockfill I and rockfill II are smaller than the test parameters while those of gravelly clay are larger than the test parameters. Overall, the model parameters are relatively low. The possible reasons may be that (1) the breakage of rockfill particles due to compaction and the rainfall infiltration during the construction period cause the softening of rockfill materials; (2) the creep and wetting deformation is not considered, which corresponds to a reduction of model parameters. In regard to verifying the back-analyzed model parameters, Figure 7 shows the comparison between calculation results and observation data at two typical measurement points, that is, DB-C-V-04 (vertical displacement) and DB-C-H-05 (horizontal displacement), and the mean absolute error (MAE) and root mean square error (RMSE) between the simulated and observed results were calculated and marked in the figure. It can be seen that good agreement is indicated.

#### 4.2.2. The 2nd Displacement Back-Analysis

To investigate the effects of creep and wetting deformation, the creep and wetting model parameters, as well as the two main E-B model parameters (*K*, *K*_*b*_), were back-analyzed during the 2nd displacement back-analysis. Here, the two E-B model parameters *m* and *n*, with less influence, took fixed values determined by considering both test and previous back-analysis results. For the seven parameters of the creep model adopted, the parameters *m*_*c*_, *n*_*c*_, and *L*_*c*_, with less variation according to engineering experiences, took fixed values determined by test, and the parameters *b* and *β*, both describing volume deformation, changed proportionally. Therefore, together with  *α* (creep rate) and *d* (reflecting shear deformation), there were three independent creep model parameters for back-analysis, whereas for the three parameters in the wetting model, if *a*_*w*_ and *b*_*w*_, both describing volume deformation, change proportionally, it results in two independent model parameters where *c*_*w*_ reflects shear deformation. Owing to the fact that the variation of upstream water level before December 2011 is small, the observation data before December 2011 were used to back-analyze the E-B and creep model parameters, and the observation data afterwards were used to back-analyze the wetting model parameters.

**Figure 8:**
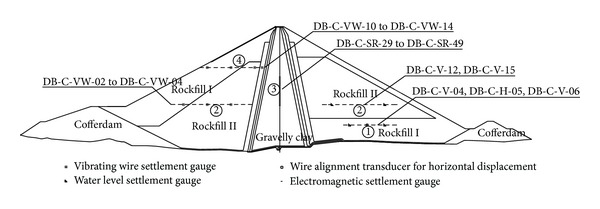
The measurement points used in the 2nd back-analysis.

The E-B and creep model parameters of the three main dam materials were decoupled as before. The locations of the measurement points used in the 2nd back-analysis are shown in Figure 8. The model parameters of rockfill I were back-calculated from the observation data of DB-C-H-05, DB-C-V-04, and DB-C-V-06. The model parameters of rockfill II were back-calculated from the observation data of DB-C-VW-02, DB-C-VW-03, DB-C-VW-04, DB-C-V-12, and DB-C-V-15. In addition, the model parameters of gravelly clay were back-calculated from the observation data of DB-C-SR-29 to DB-C-SR-49. The back-calculated parameters of the 2nd back-analysis, as well as the test parameters, are listed in Table 3. From Table 3, it can be seen that the back-calculated *K* and *K*_*b*_ are larger than the test parameters, which, in a sense, verifies the second reason for the lower model parameters in the previous back-analysis. The creep rates of rockfills I and II are approximately half of the test parameters, and the volume deformation parameters are larger, whereas the shear deformation parameters are comparable. The creep model parameters of gravelly clay are comparable with their test counterparts.

The wetting model parameters of rockfill I and rockfill II were back-analyzed together, and the measurement points used in the back-analysis of wetting model parameters are DB-C-VW-10, DB-C-VW-11, DB-C-VW-12, and DB-C-VW-13 (see Figure 8). The results are shown in Table 4, from which it can be seen that the volume deformation parameters are approximately half of the test parameters, whereas the shear deformation parameters are more than twice that of the test parameters.

**Figure 9:**
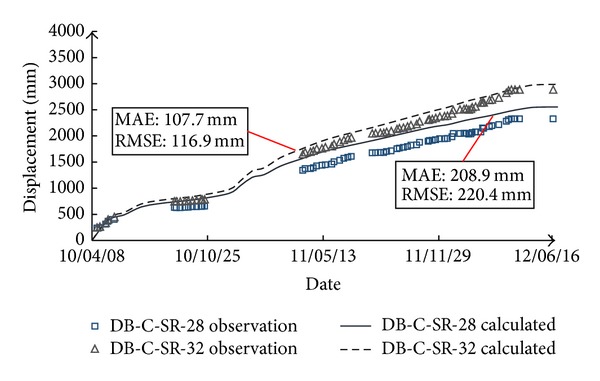
Comparison between calculated results (2nd back-analysis parameters) and observation data.

Through field examination, it was found that the compaction degree of the gravelly clay is generally better than the designed value, which, to a certain degree, justifies the high deformation moduli obtained by the back-analyses. In Figure 9 and Table 5, the results of further verification of the back-analyzed model parameters are shown, representing the comparison between calculation results and observation data at two typical measurement points. The calculation results with back-analyzed parameters are much closer to the observation data than those with test parameters.

#### 4.2.3. Analysis Based on Back-Calculated Parameters

**Figure 10:**
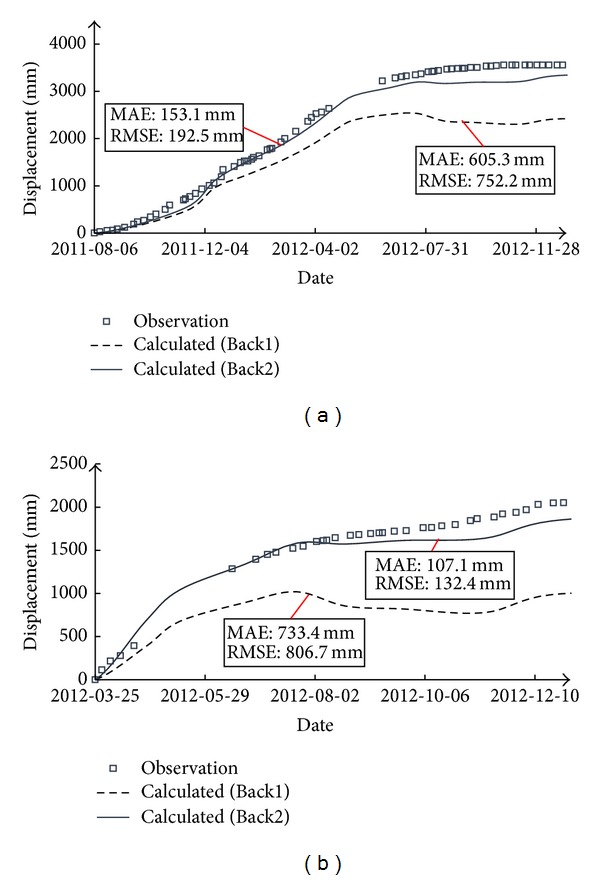
Comparison of calculation results based on the two sets of back-calculated parameters through construction completion.

In the stepwise back-analyses of Nuozhadu dam, the creep and wetting deformations were not considered in the first analysis. Although the calculation results based on the first back-analyzed parameters agree well with the observation data before impounding, the trends of calculated displacements can be different from that of the observation data in the later period. To illustrate the influence of creep and wetting deformations, Figure 10 compares the calculation results based on Back 1 model parameters without creep and wetting deformations and the results based on Back 2 parameters with creep and wetting deformations at two typical measurement points, that is, DB-C-SR-53 and DB-C-SR-63, through the end of construction. It can be seen that the calculation results of the 2nd back-analysis are much closer to the actual measurements, especially after December 2011 (impoundment of the dam started). The reason may be that the model parameters of dam materials change with time because of the breakage and wetting of rockfill particles. That is to say, the breakage of particles and impounding will cause certain deformation. Therefore, it is necessary to take the effects of creep and wetting into consideration in the numerical analyses of earth dams.

**Figure 11:**
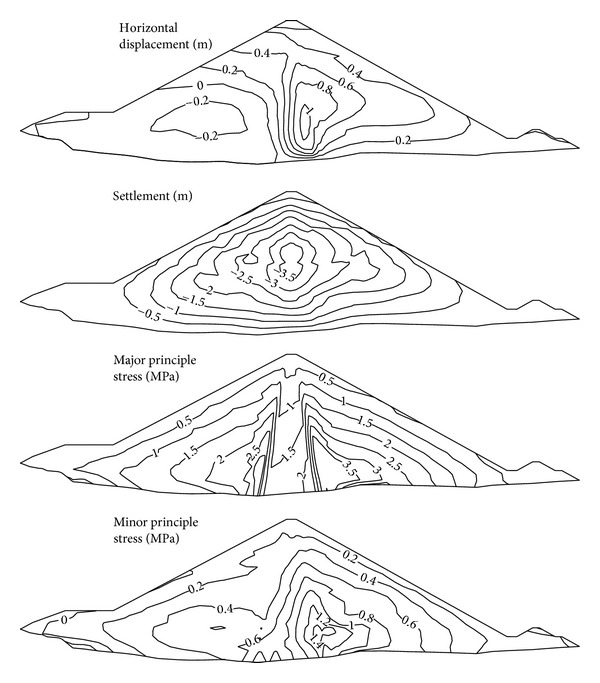
Displacement and stress distribution at completion.

With the back-calculated model parameters, the displacement and stress distributions at completion were predicted (Figure 11). The maximum horizontal displacement is 111 cm, pointing to the downstream. The maximum settlement is 384 cm, approximately 1.47% of the maximum dam height, located at the lower middle of the maximum cross-section. The overall stress distribution agrees with the general distribution of earth-core rockfill dams and displays a clear arch effect. Due to buoyancy, water pressure, and large permeability differences between the rockfill and core wall, the maximum stress occurs at the bottom corner of core wall and downstream rockfill zone.

## 5. Conclusion

The deformation observation data of the Nuozhadu high earth-rockfill dam, which fully reflects the state of the dam, plays an important role in analyzing the characteristics of the dam materials and facilitating the prediction of future deformation. In this study, the model parameters of Duncan and Chang's E-B model, the seven-parameter creep model, and a modified Shen's three-parameter wetting model of the Nuozhadu high earth-rockfill dam were back-analyzed based on field monitoring displacement data by employing an intelligent back-analysis method. Two displacement back-analyses have been performed at different construction stages, with and without considering the creep and wetting deformations. To avoid simultaneous back-analysis of many parameters, the model parameters of the three main dam materials are decoupled and back-calculated separately according to material zoning, construction process, and observation point locations. The resulting numerical data are in good agreement with the monitoring data for most observation points. The deviation of the model parameters from the laboratory tests revealed by the stepwise back-analyses has been partially verified by field examination results. The back-analysis method and decoupling method used in the back-analysis were effective at addressing complex problems with multiple models and parameters. The comparison of calculation results based on different sets of back-calculated parameters indicates that the breakage of particles and impounding will cause certain deformation, and it is necessary to take the effects of creep and wetting into consideration in the numerical analyses of high earth-rockfill dams. With the back-calculated parameters, the stress and deformation distributions at completion were predicted and analyzed, from which conclusive results were obtained.

## Figures and Tables

**Table 1:** Stepwise displacement back-analyses.

Back-analysis	Observation data date	Upstream water level	Parameters analyzed
1	Dec. 15, 2011	666 m	*K*, *n*, *K*_*b*_, *m* (E-B)

2	May 14, 2012	738.3 m	*K*, *K*_*b*_ (E-B) *α*, *λ*, *d* (creep) *a*, *b*, *c* (wetting)

**Table 2:** Main E-B model parameters of main dam materials.

Material	*K*	*K*_*b*_	*n*	*m*
Rockfill I				
Test	1425	540	0.26	0.16
Back 1	1246	411	0.14	0.11
Rockfill II				
Test	1400	620	0.17	0.05
Back 1	1188	393	0.145	0.043
Gravelly clay				
Test	320	210	0.48	0.26
Back 1	368	244	0.226	0.038

**Table 3:** Main E-B and creep model parameters.

Material	*K*	*K*_*b*_	*α*	*λ*	*d*
Rockfill I					
Test	1425	540	0.00600	1.00	0.00423
Back 2	1486	665	0.00314	1.61	0.00311
Rockfill II					
Test	1400	620	0.00600	1.00	0.00612
Back 2	1643	717	0.00300	2.17	0.00821
Gravelly clay					
Test	320	210	0.00300	1.00	0.00717
Back 2	510	340	0.00345	1.27	0.00849

*Note*. *λ* is the scale of parameters *b* and *β*.

**Table 4:** Wetting model parameters.

Parameters	Rockfill I			Rockfill II		
	*a*_*w*_	*b*_*w*_	*c*_*w*_	*a*_*w*_	*b*_*w*_	*c*_*w*_
Test	2.820	1.730	0.362	2.980	1.780	0.356
Back 2	1.417	0.869	0.904	1.493	0.892	0.890

**Table 5:** Comparison of displacement values (mm).

Observation point	Observed	Calculated	
		Back	Test
DB-C-V-04	217.0	222.2	192.8
DB-C-VW-03	390.0	393.2	335.2
DB-C-H-05	198.9	203.4	168.6
DB-C-SR-31	664.2	698.6	830.2
